# An optimized histological proceeding to study the female gametophyte development in grapevine

**DOI:** 10.1186/s13007-020-00604-6

**Published:** 2020-05-01

**Authors:** P. Moreno-Sanz, E. D’Amato, A. Nebish, L. Costantini, M. S. Grando

**Affiliations:** 1 Center Agriculture Food Environment (C3A), University of Trento, Via. E. Mach 1, 38010 San Michele all’Adige, Italy; 2 Research and Innovation Centre, Fondazione Edmund Mach, Via E. Mach 1, 38010 San Michele all’Adige, Italy; 3 Department of Physics, University of Trento, Via Sommarive 14, 38123 Povo, Italy; 4 Department of Genetics and Cytology, Yerevan State University, 1 Alex Manoogian St., 0025 Yerevan, Armenia; 5 Research Group of Plant Genetics and Immunology, Institute of Molecular Biology NAS RA, 7 Hasratyan St., 0014 Yerevan, Armenia

**Keywords:** Grapevine, Megasporogenesis, Megagametogenesis, Paraffin-embedding, Embryo sac, Seedlessness

## Abstract

**Background:**

Reproductive success in seed plants depends on a healthy fruit and seed set. Normal seed development in the angiosperms requires the production of functional female gametophytes. This is particularly evident in seedless cultivars where defects during megagametophyte’s developmental processes have been observed through cytohistological analysis. Several protocols for embryo sac histological analyses in grapevine are reported in literature, mainly based on resin- or paraffin-embedding approaches. However their description is not always fully exhaustive and sometimes they consist of long and laborious steps. The use of different stains is also documented, some of them, such as hematoxylin, requiring long oxidation periods of the dye-solution before using it (from 2 to 6 months) and/or with a differentiation step not easy to handle. Paraffin-embedding associated to examination with light microscope is the simplest methodology, and with less requirements in terms of expertise and costs, achieving a satisfactory resolution for basic histological observations. Safranin O and fast green FCF is an easy staining combination that has been applied in embryological studies of several plant species.

**Results:**

Here we describe in detail a paraffin-embedding method for the examination of grapevine ovules at different phenological stages. The histological sample preparation process takes 1 day and a half. Sections of 5 µm thickness can be obtained and good contrast is achieved with the safranin O and fast green FCF staining combination. The method allows the observation of megasporogenesis and megagametogenesis events in the different phenological stages examined.

**Conclusions:**

The histological sample preparation process proposed here can be used as a routine procedure to obtain embedded ovaries or microscope slides that would require further steps for examination. We suggest the tested staining combination as a simple and viable technique for basic screenings about the presence in grapevine of a normally and fully developed ovule with embryo sac cells, which is therefore potentially functional.

## Background

Plant histological proceedings include a wide variety of methods and techniques that can be used in different combinations to achieve the desired result. Most of the currently used plant microtechniques were developed in the first half of the 20th century, when this science was strongly driven [1–4]. However, the introduction of the polymerase chain reaction (PCR) [5] in the last decades of the century put the focus on molecular biology. The need of molecular biologists to visualize a gene or gene product in the context of the whole plant, or to verify hypothesized defective ontogenesis, morphogenetic events and structural changes, has relaunched the interest in plant microtechniques combined with microscopic observations [6]. A good histological study based on anatomical and histochemical alterations provides insight into cellular processes and gives clues to propose hypotheses for further experimentation. In fact, more and more researchers incorporate cytohistological analyses into their investigations because it allows to “see” changes that take place in the target experimental system [7–15]. Plant tissue culture methods are often applied to fundamental studies of plant morphology and development. Such studies demand familiarity with histological techniques for light microscopy. Observation and, specially, interpretation of the histological sections usually requires a high degree of expertise and a steep learning curve that can be facilitated by the use of specific plant anatomy atlas [16]. Nevertheless, the technical procedure to obtain the histological sample can be easily reproduced if tissue-specific well-described protocols are available.

There is a vast literature on plant microtechniques and microscopy proposing a broad collection of protocols with general schedules that have to be optimized depending on the species, the nature of the tissue and the aim of the experiment; therefore each schedule has to be adjusted for every histological experiment to be performed, so that a high quality histological sample could be obtained in the shortest possible time. The availability of *ad*-*hoc* schedules for a certain tissue of a determined species supposes a considerable saving of time. It also becomes of great value especially for researchers with limited knowledge and experience in this field, but whose research topic requires the application of histological procedures to keep moving forward in their investigation.

Three main parts can be identified in the histological sample preparation. Part 1: sample preparation for sectioning, resulting in the tissue embedded in a solid matrix ready to be sectioned. It usually includes the following steps: fixation, dehydration, clearing, infiltration and embedding. Part 2: sectioning and affixing to microscope slides to obtain good quality sections in terms of thickness (the thinner the better resolution). Part 3: staining of the obtained sections for visualization at the light microscope (see [1–4, 6] for detailed description of each step).

Detailed knowledge of the reproductive biology of cultivated species is important, not only to assess the adaptive significance and homology of descriptive characters used in plant systematics, but also to comprehend the requirements for fruit and seed production that allow the development of effective management strategies and a sustainable use [17]. Reproductive success in seed plants depends on a healthy seed set [18]. Normal seed development in the angiosperms requires the production of functional male (pollen grain or microgametophyte) and female (embryo sac or megagametophyte) gametophytes [19]. This is particularly evident in seedless cultivars where defects during megagametophyte’s developmental processes have been observed through cytohistological analyses [14, 20–22]. Unlike microgametophyte’s phenotyping (which can be subjected to high-throughput phenotyping for its functional validation [23]), there is a technical difficulty in obtaining phenotypic information from the megagametophyte at the cellular or sub-cellular level, as it is a more complex system and is located within the ovule and wrapped by the integuments, which act as physical barriers [24].

A complete functional embryo sac is critical to many steps of the reproductive process, so a deep comprehension of female sporogenesis (megasporogenesis) and gametogenesis (megagametogenesis) is needed to further understand other processes of the reproductive biology in which the megagametophyte is involved, such as pollen tube guidance, fertilization, induction of seed development upon fertilization, and maternal control of seed development after fertilization [25, 26]. In grapevine, the female reproductive organ (gynoecium or pistil) consists of a superior ovary with two locules (each containing two anatropous ovules with an embryo sac), a single short style and a single stigma. The ovule, which develops as a placental outgrowth, is constituted by a massive nucellus and two integuments (inner and outer). The nucellus evolves from periclinal divisions of the subepidermal cells of the ovule primordium. The inner integument originates at the base of the ovule primordium from periclinal divisions of the nucellar epidermal cells and consists of two to three cell layers. The outer integument, instead, develops at the base of the inner integument from periclinal divisions of the subepidermal cells when the ovule is partly anatropous and, in this case, consists of two to nine cell layers in different parts of the ovule [27]. Embryo sac development starts with differentiation of one hypodermal cell in the center of the nucellus into the archeosporial cell (2n), which divides transversely and forms an outer primary parietal cell (from which the layers of parietal cells and nucellar calotte generate) and an inner primary megasporogenous cell [27, 28]. During megasporogenesis, the inner primary megasporogenous cell differentiates into the megaspore mother cell (MMC), which undergoes two consecutive meiotic divisions producing a linear tetrad of four megaspores (n). Only the one in the chalazal direction behaves as a functional megaspore and develops into the female gametophyte, whilst the remaining meiotic products degenerate by a form of programmed cell death. During embryo sac development the ovule becomes anatropous (completely inverted) due to the development of the integuments. Then, during megagametogenesis, the surviving megaspore undergoes three consecutive mitosis resulting in an immature embryo sac that consists of an eight-nucleate cell (four nuclei toward the micropyle and another four toward the chalazal end). Finally, a last differentiation process gives rise to a mature monosporic *Polygonum*-type embryo sac containing seven cells (three antipodals in the chalazal end, the egg apparatus consisting of two synergids and one egg cell in the micropyle end, all of them haploid, and one homodiploid central cell near the egg apparatus) [25, 27, 29, 30]. Lebon et al. [31] studied the reproductive organ development in two grapevine cultivars (Gewürztraminer and Pinot Noir) applying a resin-embedded approach. These authors observed that, in both cultivars, female reproductive cells consisted of sporogenous tissue between stages E-L 12 and E-L 15 [32], reaching the MMC stage at E-L 15 + 2 days. The time course of female development differed thereafter. Residual megaspore generating the embryo sac was formed at E-L 15 + 8 days in Pinot Noir and E-L 17 in Gewürztraminer. However, at the onset of anthesis the embryo sac was fully developed in both cultivars.

In literature there are several works where cytohistological approaches have been applied for the study of the embryo sac in grapevine as well as for the investigation of flower and berry development [14, 21, 22, 33–39]. The various methodologies are mainly based on resin- or paraffin-embedding techniques associated with electron (EM) or light microscopy (LM), respectively. In resin-embedding techniques ultrathin sections (1 nm) can be obtained from very hard material (deep freezing or resins) providing high resolution images at the EM; however these kind of techniques require a high level of expertise and special microtomes designed for obtaining very thin sections [34]. Unlike resin-based techniques, paraffin-embedding approaches contain the costs and, despite that the best resolution achieved is 3–5 µm, they can be an efficient tool if there is no need to observe ultrastructural features of small regions or even of a particular cell type [21, 22, 36, 39]. In addition, it is easier to orient and embed specimens in wax than in polyester wax or glycol methacrylate. In some cases, a combination of resin-embedding with LM, instead of EM, has also been applied in order to get semi-thin sections (1 µm), an intermediate approach in terms of expertise, costs and image resolution [14, 31]. However, in the published protocols the description of the used cytohistological methodology is not always exhaustive.

Different staining procedures have been performed depending on the target structures or compounds to be observed. There is a huge variety of available stains in botanical microtechnique, but hematoxylin still remains the standard nuclear stain for histological studies. However, hematoxylin by itself is a very weak dye and is of no value in microtechnique if it is not used in conjunction with a mordant that causes it to act as a very strong basic dye. Safranin O and fast green FCF staining combination, easier to apply than hematoxylin, is also considered one of the most valuable stains both for nuclear as well as anatomical and embryological studies and it has even replaced hematoxylin stain for routine work in some cases [40]. This staining combination has been applied in embryological studies of several plant species [9, 41, 42] and, in grapevine, as far as we know, for carpel morphogenesis, flower formation, fruit set and early stages of fruit development studies [37, 38, 43].

Here we present a detailed paraffin-embedding method for the examination of grapevine ovules at different phenological stages. The incubation times at each step (fixation, dehydration, clearing, infiltration, and staining) have been optimized in order to reduce the duration of the entire process. The main objectives were to develop optimized schedules for: (i) preparing good quality histological samples of grapevine ovules for further processing (part 1 and 2 of the histological sample preparation process, see above) and (ii) testing the safranin O and fast green FCF staining combination as a valid method for routine screening of functional embryo sacs in grapevine.

## Results

The histological proceeding proposed here (consisting in dehydration, clearing, infiltration and embedding steps) takes 1 day and a half, allowing the preparation of 12 samples at the same time (more samples can be processed simultaneously if the volume of the solvents used at each step is increased). Cooling the paraffin block with ice for 1–2 min prior sectioning (i.e. holding one ice cube against the paraffin block so that the melting ice water will drop down over its front surface) is useful to obtain thin sections. Anyway, despite the technical feasibility of obtaining good sections of 3 µm thickness with the rotary microtome, it was difficult to place them into the paraffin section flotation bath, as they tended to fold. Therefore, sections of 5 µm thickness were preferentially produced.

### Analysis of the megagametophyte development

**Fig. 1:**
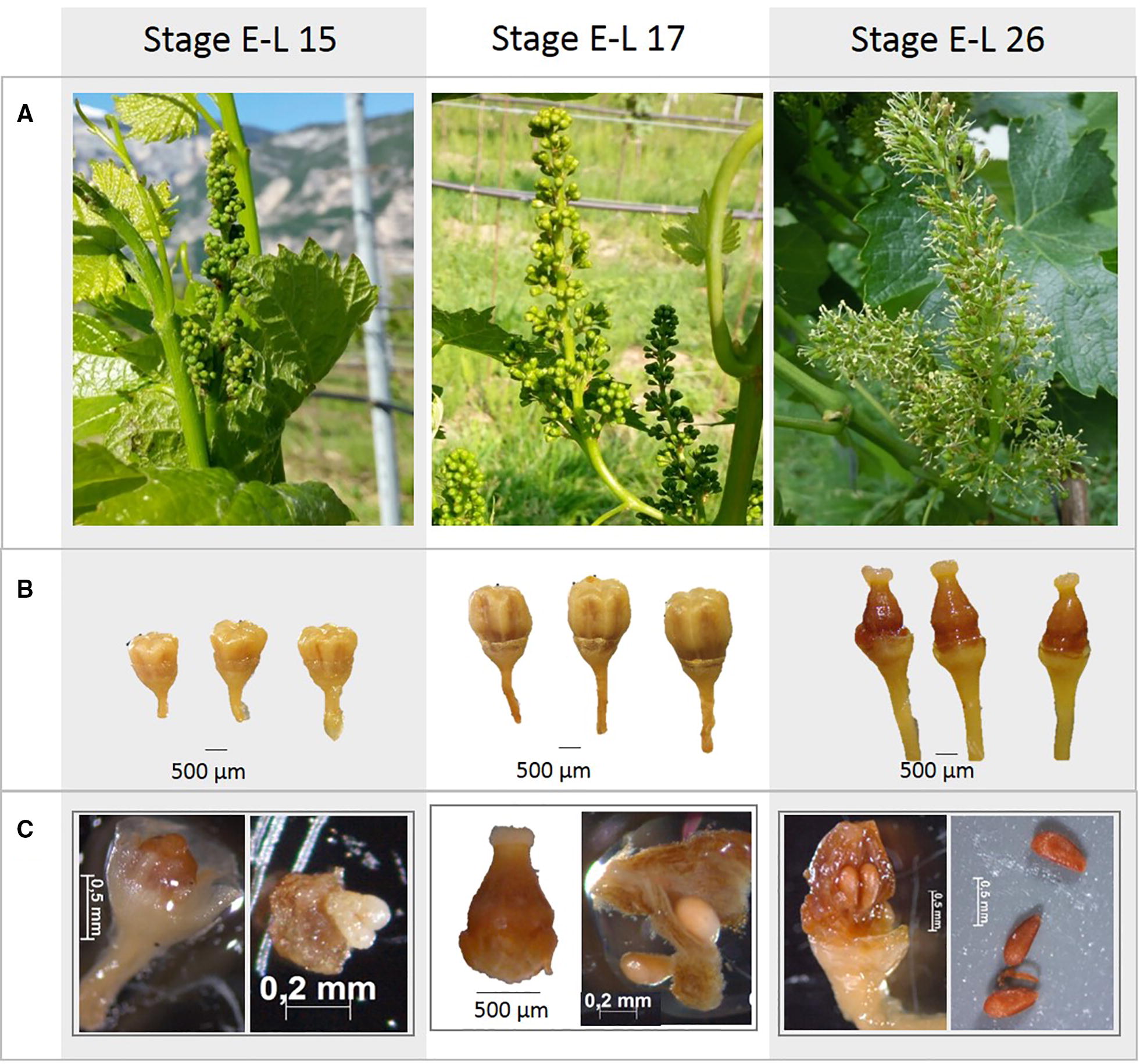
Inflorescences (**A**) and flowers, pistils and ovules fixed on FAA (**B**, **C**) at the three phenological stages examined: E-L 15, E-L 17 and E-L26

Sangiovese ovules at three phenological stages were examined in both longitudinal and transversal orientations (Fig. 1, Table 1):

**Table 1:** Summary of the histological observations in the different flowers and ovules from Sangiovese cv. examined at three phenological stages

Stage	Orientation	Ovules	Inner Integument	Outer Integument	Nucellus	Embryo sac elements	Special observations
E-L 15	Longitudinal	Beginning stages of ovule development	Initiation of inner integument	Signs of initiation of periclinal divisions of cells at the base of the inner integument	Nucellus is developing, consists of 2–3 layers of cells	Archaeospore, mitosis of archaeospore	Archaeospore is in the central part of the nucellus, in some ovules mitosis of archaeospore is observed
E-L 17	Longitudinal	All ovules are anatropous	Developed inner integument, three layers of cells	Developed outer integument. Four to seven layers of cells in different parts of the ovule	Normally developed	Surviving megaspore after meiosis of MMC. In some ovules megagametogenesis during the first or second mitosis	There is a small space for processes of megasporogenesis in the centre of nucellus
E-L 17	Transversal	Ovaries mainly constituted by two locules and four ovules, rarely by three locules and six ovules	Developed inner integument, three layers of cells	Developed outer integument. Four to seven layers of cells in different parts of the ovule	Normally developed	Megaspores	In some ovules there is a narrow space between the inner and the outer integument
E-L 26	Longitudinal	Developed ovules with cells of embryo sac or degenerating ovules without embryo sac elements	Developed inner integument, three layers of cells	Developed outer integument. Four to seven layers of cells	Normally developed	Mature embryo sac with central cell, synergids and egg cell	In some ovules there is a space between the inner and outer integuments, sometimes between inner integument and the nucellus even in ovules with cells of embryo sac. Exfoliated integuments are the first sign of ovule degeneration
E-L 26	Transversal	Ovaries mainly constituted by two locules and four ovules, rarely by three locules and six ovules. Elements of fertilization process undertaking	Developed inner integument, three layers of cells	Developed outer integument. Four to seven layers of cells	Normally developed	Cells of the embryo sac separately in different sections: central cell or synergids and egg cell. Evidences of fertilization process: zygote, endosperm, sperm cell	There is a space between the inner and outer integuments in the majority of ovules. In some slides a small space between integuments and/or nucellus could be due to microtome damaging because microtome tearing was also observed in these slides

**Fig. 2:**
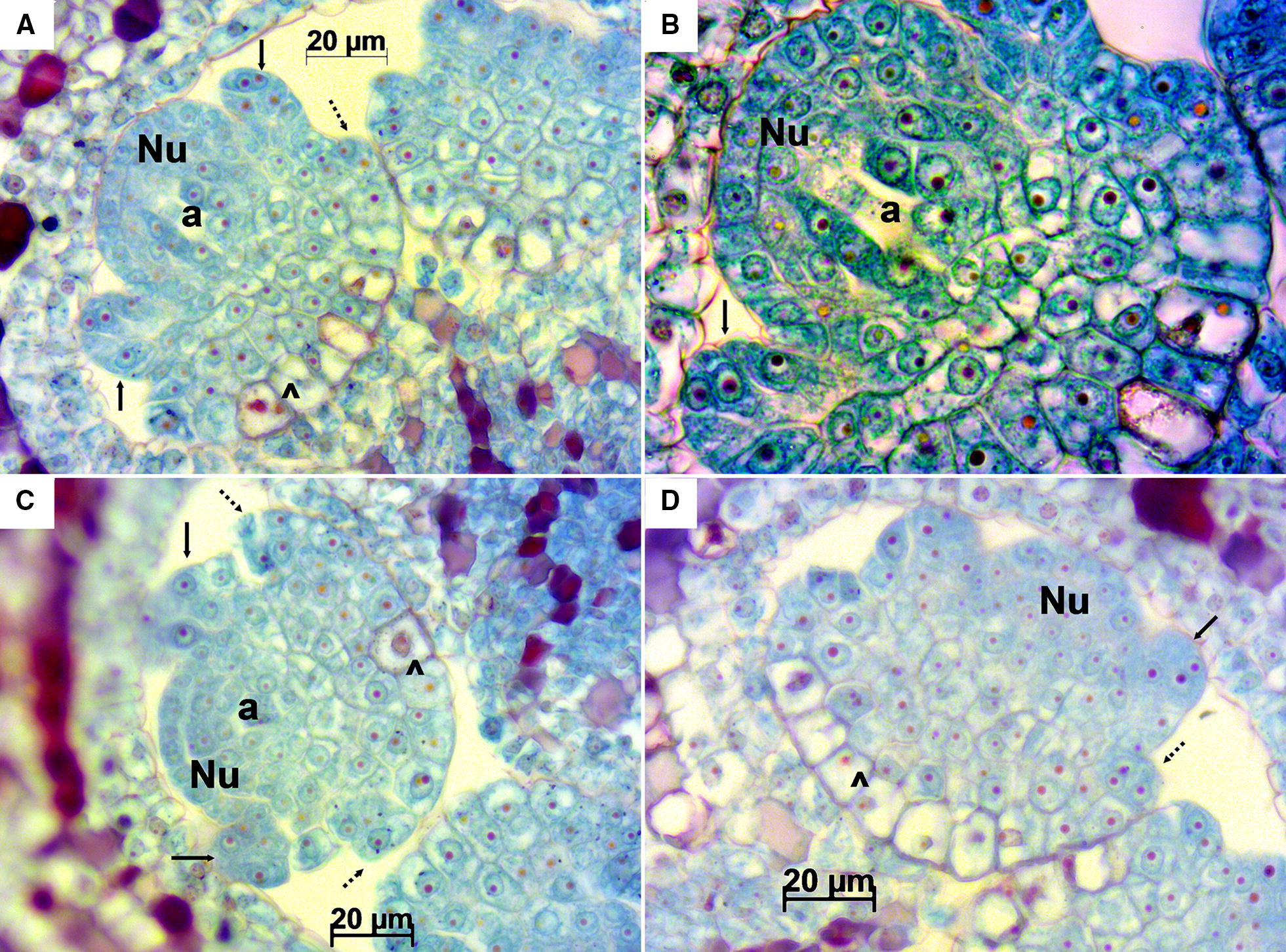
Longitudinal sections of Sangiovese ovules at stage E-L 15. Magnifications: 400× in **A**, **C** and **D**, 1000× in **B**. In **A** and **B** the empty space in the archeospore (a) in the central part of the nucellus (Nu) shows the process during metaphase of mitotic division of the archeospore (a) which will produce an outer primary parietal cell and an inner primary megasporogenous cell. In **C** the archeospore (a), in the central part of the nucellus (Nu), is the largest cell with a little darker area. Initiation of the inner integument (black arrow), dividing cells (^) indicating signs of initiation of the outer integument formation (dotted arrow)
- Stage E-L 17.
  Longitudinal ovules at this stage were already anatropous with nucellus and both integuments fully developed (Fig. 3A, B). Transversal sections offered an overview of the ovary, which was mainly divided in two locules with two ovules each, accounting a total of four ovules per ovary. However, in some cases, ovaries had three locules with two ovules each, thus containing an overall of six ovules with a megaspore each (Fig. 4). Inner and outer integuments completely wrapped the nucellus in all the ovules examined. Inner integument consisted in a three cell layer and outer integument in four layers of cells reaching seven in the chalazal part of the ovule. Nucellus and inner integument cells appeared cyan/green with reddish/magenta nuclei, while the outer integument cells presented a more intense safranin O staining (not only in the nuclei and nucleoli), especially those in the outermost layer of the outer integument. A space between the inner and outer integument was observed in the majority of ovules and sometimes between the nucellus and the inner integument, as well as exfoliated integuments (Fig. 4A–F, H). The nucellar calotte or nucellar cap, which develops at the mycropilar end of the nucellus, consisted at this stage of around five layers of cells (Fig. 3B, E). Different processes that take place during megagametogenesis were observed at this stage (Figs. 3 and 4B–E, Table 1) with megaspores during or after the first or second mitosis.

- Stage E-L 15.
  Longitudinal sections of Sangiovese ovules were obtained at this stage. In all the investigated ovules the beginning of the nucellus was observed as well as protuberances indicating the initiation of the inner integument. The archeospore was evident in most of them. Cells undergoing periclinal divisions at the base of the inner integument were visualized, which likely point to an initial formation of the outer integument. The cytoplasmatic component of the cells forming the nucellus and the observed protuberances were stained with cyan/green hues while nuclei and nucleoli were clearly stained in reddish/magenta (Fig. 2).

**Fig. 3:**
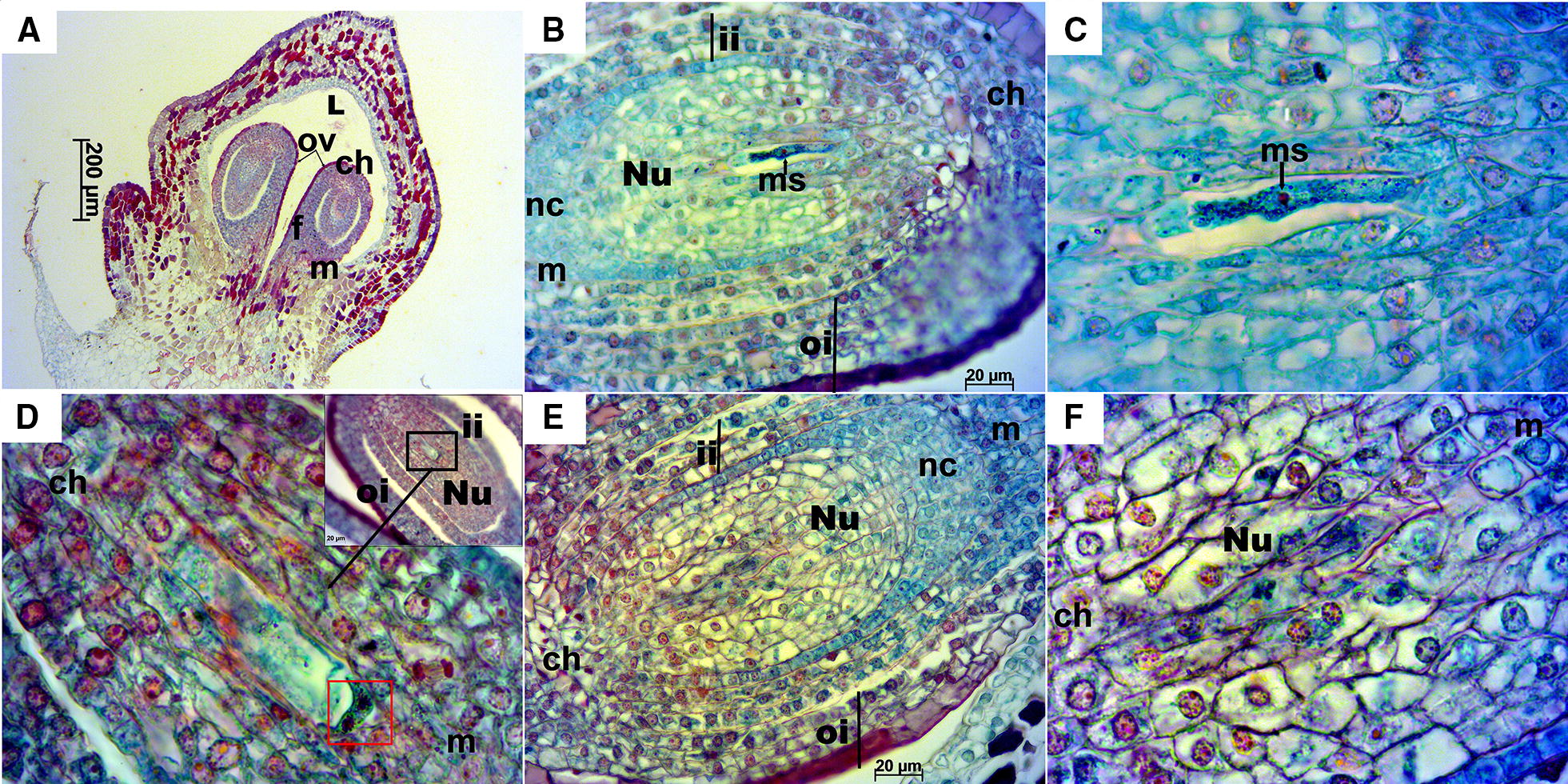
Longitudinal sections of Sangiovese ovules at stage E-L 17. **A** Anatropous ovules at 50×. **B** Ovule with megaspore at 400× in the stage of two nuclei after first mitosis. **C** Detailed megaspore in **B** at 1000×. **D** Megagametogenesis during the second mitosis, in the stage after anaphase and near telophase (red box), at 1000× with the ovule overview in the right upper corner of the image. **E** Ovule with well-developed nucellus around embryo sac, inner and outer integuments. **F** Well-developed nucellus in the ovule. ch: chalaza, f: funiculus, ii: inner integument, L: locule, m: micropyle, ms: megaspore, nc: nucellar cap, Nu: nucellus, oi: outer integument, ov: ovule

**Fig. 4:**
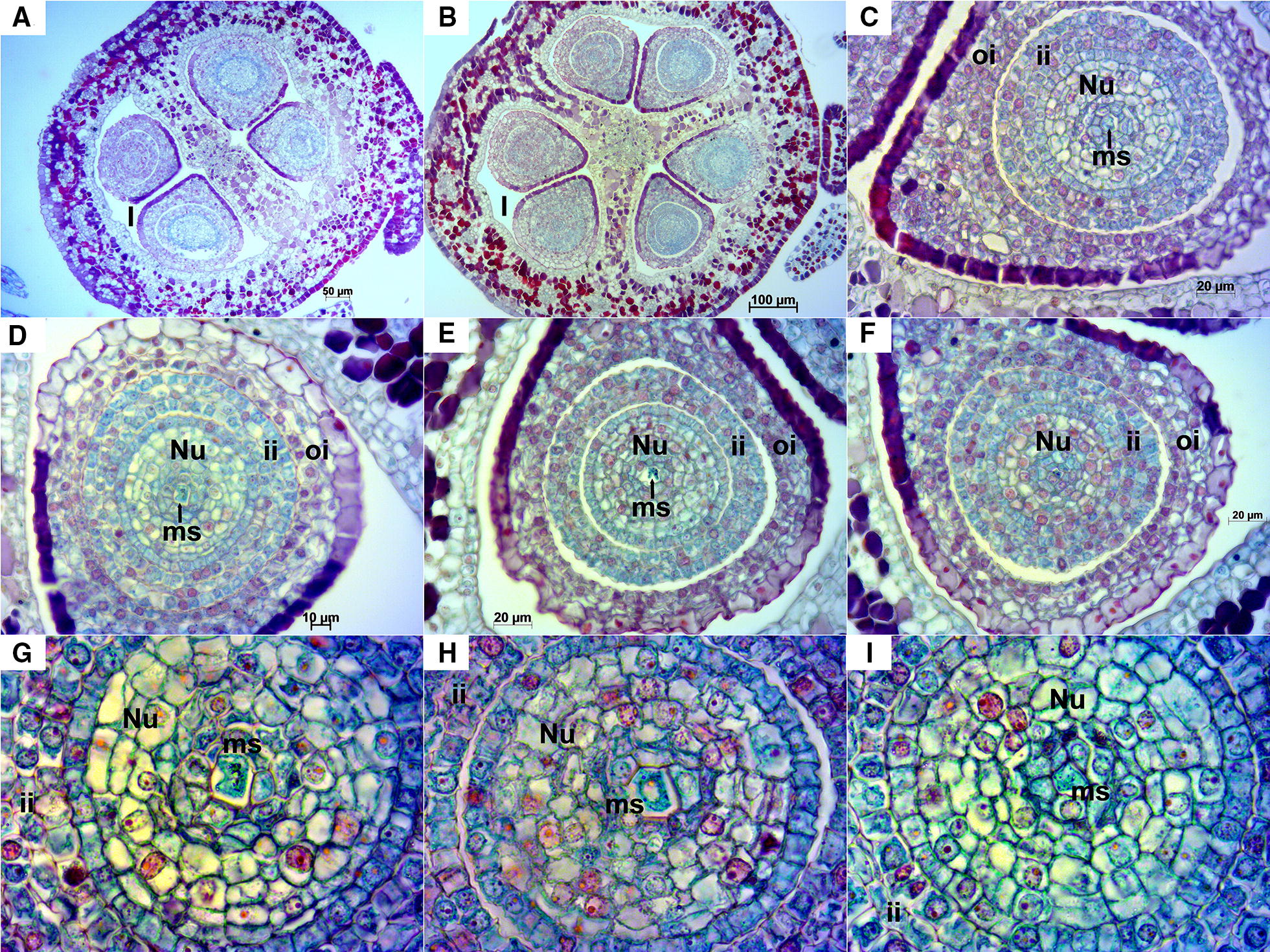
Transversal sections of Sangiovese ovaries and ovules at stage E-L 17. **A**, **B** Transversal section at 100× of the ovary of two Sangiovese flowers presenting two (**A**) or rarely three locules (**B**) with two ovules each one. **C**–**F** sections of ovules containing the megaspore during starting mitosis at 400×. **G**–**I** megaspore in each section of ovules in the central part of nucellus at 1000×. Nu: nucellus, l: locule, ii: inner integument, oi: outer integument, ms: megaspore
- Stage E-L 26.
  Longitudinal ovules examined at this stage were anatropous with fully developed integuments (Fig. 5). In transversal sections, as in stage E-L 17, some ovaries with three locules containing two ovules each were observed; in this case most of them presented a mature embryo sac (Fig. 6). Anyway, the examined ovaries consisted mainly of two locules. A small space could be appreciated between the inner and outer integuments (Figs. 5B, D, 6A–C, E, G–L) and less frequently between the nucellus and the inner integument (Figs. 5B, 6C). At this stage cell layers of both inner and outer integuments stained with a reddish/magenta color; although, as in stage E-L 17, cells of the outermost layer of the outer integument presented a much more intense staining compared to the other layers. The nucellar cap consisted of up to ten layers of cells. Embryo sac elements (egg cell, synergids and central cell or its contours) could be seen in the majority of the examined ovules in both longitudinal (Fig. 5) and transversal sections (Fig. 6). In addition, in some ovules the zygote, the endosperm and/or the sperm cell were visible, which indicates that the fertilization process was already undertaken (Fig. 6E–G, I–K). Nucellar tissue was not observed in two ovules from the same flower in any of the longitudinal sequential sections inspected (Fig. 5A).

**Fig. 5:**
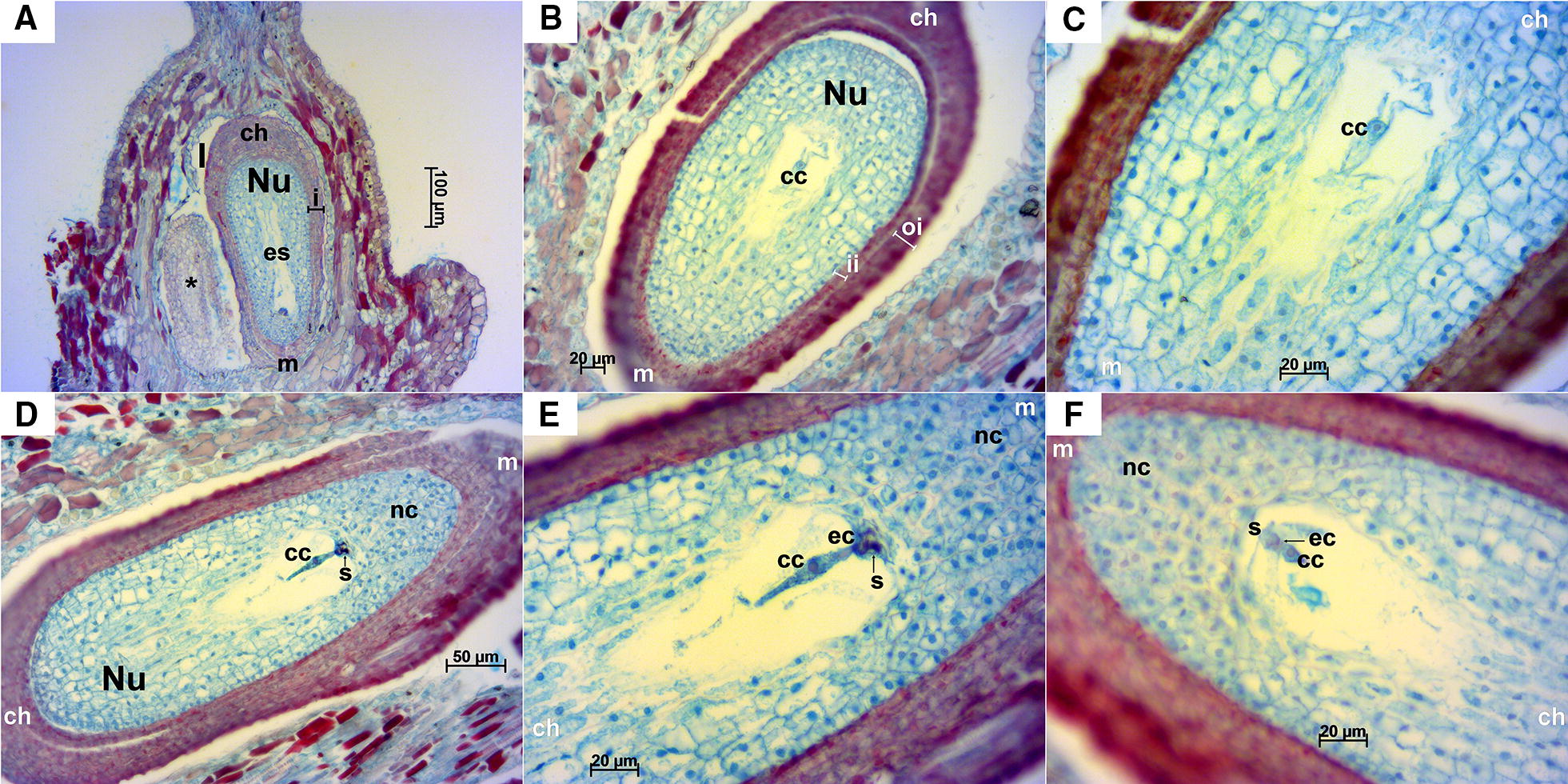
Longitudinal sections of Sangiovese ovules at stage E-L 26 (flowering). **A** Two ovules within one locule of the ovary at 100×. In the left a small-sized ovule (*) where no nucellus cells nor cells of embryo sac were visible. Integuments (i), nucellus (Nu) and mature embryo sac (es) can be observed in the right ovule. **B**, **C** Ovule with mature embryo sac with central cell and contours of synergids at 200× (**B**) and 400× (**C**), microtome damaging can be observed as a cut perpendicular to the outer integument in the left side. **D**, **E** Ovule with embryo sac elements: central cell, egg cell and dark synergid, at 200× (**D**) and 400× (**E**). **F** Embryo sac with egg cell, synergids and central cell at 400×. cc: central cell, ch: chalazal end, ec: egg cell, es: mature embryo sac, i: integuments, ii: inner integument, l: locule, m: micropyle end, nc: nuclear calotte, Nu: nucellus, oi: outer integument, s: synergids

**Fig. 6:**
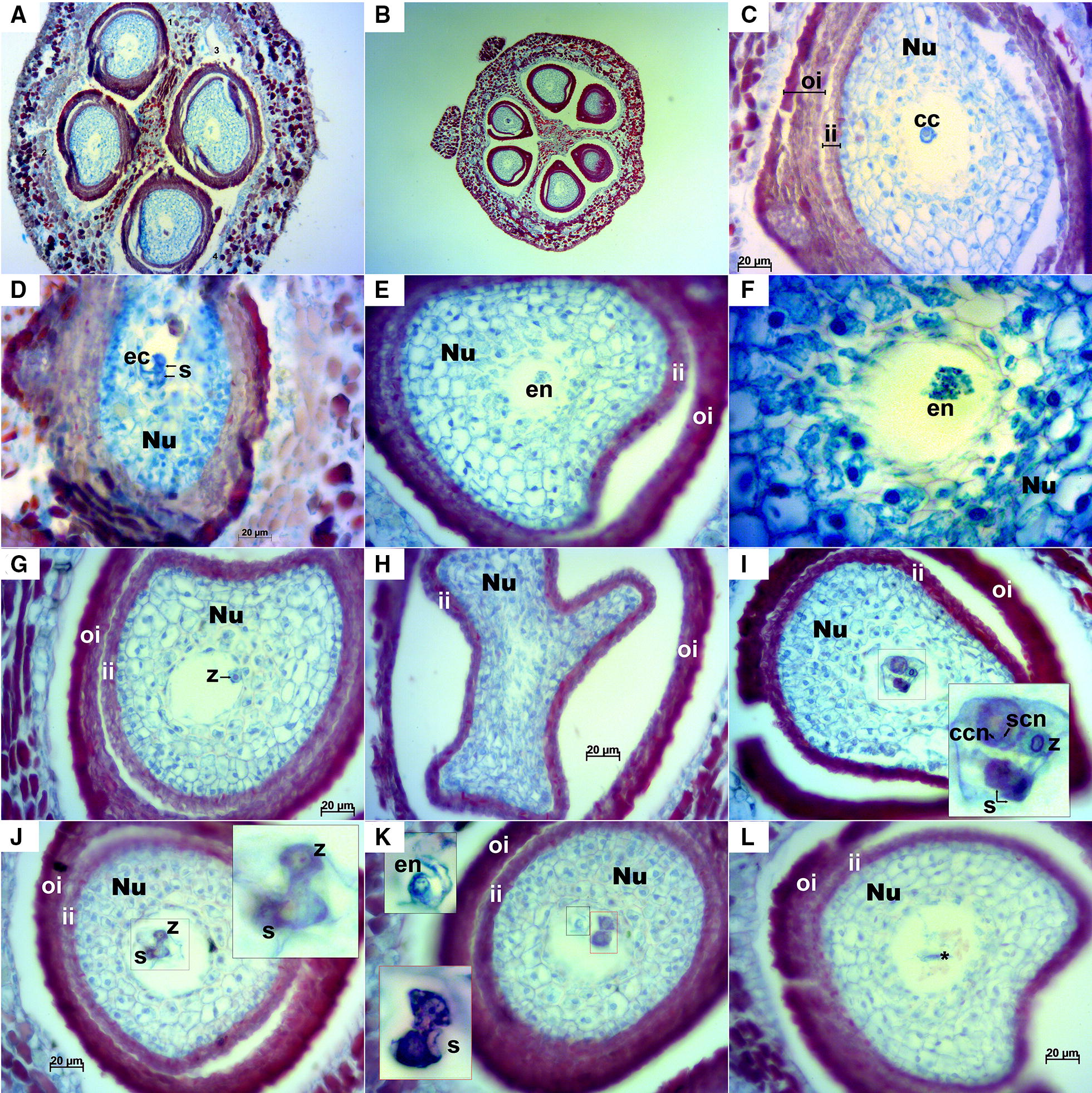
Transversal sections of Sangiovese ovules at stage E-L 26 (flowering). **A** Sangiovese ovary at 100× with two locules and four ovules with exfoliated integuments (they can partly be the result of microtome damaging). **B** Rare ovary presenting three locules with two ovules each at 50×. **C** Exfoliated integuments partly due to microtome damaging, embryo sac with central cell at 400×. **D** Egg cell and two synergids at 400×. **E**, **F** Embryo sac with initiation of the endosperm at 400× (**E**) and endosperm nuclei in detail at 1000× (**F**). **G** embryo sac with an already visible zygote at 400×. **H** one of the first transversal sections of a Sangiovese ovule at 400× with an unusual shape of the inner integument. **I** section of the middle part of the same ovule shown in **H** where the fertilization process is occurring: zygote just after fertilization by one sperm cell, two dark synergids together and a large central cell with two nuclei including nucleus of the central cell (2n) and the nucleus of the second sperm cell (n), 400×. **J** Ovule at 400× with an already visible zygote and synergids (zoom in the upper right corner). **K** Embryo sac after fertilization where the initiation of the nuclear endosperm with several micronuclei (black dotted area, zoom in the left upper corner) and two dying synergids (red dotted area, zoom in the left bottom corner) are visible, 400×. The upper synergid is at the beginning of the cell death process while the one in the bottom is already dead. **L** Embryo sac with dead cells in the center (*), 400×. cc: central cell, ccn: central cell nucleus, ec: egg cell, en: endosperm, ii: inner integument, Nu: nucellus, oi: outer integument, s: synergids, scn: sperm cell nucleus, z: zygote

## Discussion

### Technical remarks

Sectioning and affixing the sections on the microscope glass slides, in our opinion, is the real bottleneck of the whole process. Dehydration, clearing and infiltration steps can be performed for several samples simultaneously, but each block of embedded material has to be sectioned once at a time. In addition, the study of the development of the embryo sac requires the examination of a set of adjacent sections because the different target structures could be in different cutting planes. Therefore, all sections from the beginning to the end of the ovule have to be kept, which makes the process even longer. On the other hand, in order to avoid mechanical damage of the tissue, sectioning at the microtome requires competence, precision and time and these skills can only be obtained and improved with practice.

Alternate sections were selected for staining. In this way, if other types of analysis (e.g. in situ hybridization, specific staining of certain structures or compounds such as lipids, carbohydrates, polysaccharides, etc.) have to be performed in a second time, specific procedures and dyes could be applied to adjacent slides to those already screened containing the structures of interest to deepen.

### Histological sample preparation and staining

Fixation time of grapevine flower buds reported in literature ranges from 12 to 72 h, usually this process has been carried out at 4 °C and sometimes vacuum has been applied to remove air and promote penetration of the fixative [14, 20, 22, 36]. According to our experience, flower buds are completely fixed after 12 h and vacuum is only needed for closed flowers collected before anthesis. That is because the calyptra creates an air chamber around the pistil and, if this air is not removed, it can interfere, not only with fixation, but also with the successive steps of the process [4]. This step could be ignored if pre-flowering flower buds are decapped before fixation. FAA universal fixative, a mixture of formalin—acetic acid—alcohol, was chosen here because it is the most extensively used by botanists. Unlike other fixatives, FAA can also work as a preservative where tissues can be stored for an indefinite period of time [3, 4, 6]. In addition, we wanted to avoid the use of other more dangerous and toxic compounds such as chromic or picric acid. Finally, FAA fixative does not need to be washed off before the dehydration step because its ingredients are soluble in the dehydrating agents and are removed before infiltration is begun.

Instead of ethanol, an ethanol-based alcohol mixture (histoalcohol), optimized for cytohistological analysis, has been used for tissue dehydration. In the consulted literature referring to grapevine, only Cardoso et al. [36] specified the ethanol series employed. They performed six steps of increasing ethanol concentrations, from 10 to 90%, and a last overnight dehydration step in 100% ethanol. The full length of the dehydration step in the protocol proposed in the present work is much shorter: 3 h and 15 min (4 h including “clearing”). The general rule concerning the initial concentration of alcohol to be used for dehydration is to begin with approximately the same percentage of alcohol as fixative or storage fluid. In this protocol dehydration steps were saved because, as the FAA fixative is already 50% ethanol, dehydration starts at 50% [6]. As dehydration, tissue infiltration from the clearing agent to the support matrix should occur gradually. In fact, in basic protocols described in plant microtechniques’ manuals this step is laborious and long, it could even last several days [1–4, 6]. For grapevine flower buds a three-step infiltration consisting of 100% xylene, xylene:paraffin 1:1 and 100% paraffin, was reported [36]. Due to the small size of the samples analyzed in the present paper, they were placed in biopsy bags to prevent them from coming out from the biopsy cassette. These filter bags absorb and retain the solvents used, as xylene, so infiltration was performed by directly placing the samples from the clearing solution (xylene) into liquid paraffin, with one change of the liquid matrix and skipping over the step xylene:paraffin 1:1.

A vast range of dyes and stain combinations are available. Different stains can be applied to the same tissue depending on the structures to be observed. In the study of grapevine ovules different dyes (i.e. Heidenhein’s iron alum hematoxylin, DAPI, aniline blue, toluidine blue O, cresyl violet, vanillin-HCl) and double stain combinations (i.e. Mayer’s hematoxylin and eosin (H&E), safranin O and orange G) have been used with different scopes [14, 20–22, 34–36, 39]. In the present work good contrast and differential staining allowing the identification of embryo sac structures were reached using this combination. Therefore, we propose safranin O and fast green FCF as a suitable staining for this purpose.

### Grapevine female gametophyte development

Stages E-L 15, E-L 17 and E-L 26 were selected to follow the female gametophyte development in Sangiovese according to the key steps of sexual organ formation observed at different phenological stages by Lebon et al. [31]. In Sangiovese at stage E-L 15 the initiation of the inner integument was already evident, as well as a nucellus with in the middle a structure that can correspond to the first division of the archeospore or the megaspore mother cell [27]. At stage E-L 17 ovules were already anatropous and megagametogenesis processes could be observed (Fig. 3D). The acquisition by the inner and outer integuments of a reddish/magenta color is likely due to the fact that this cell layers consist of tannin-bearing cells, specially the outermost layer of the outer integument, as described by [44]. The spaces observed between inner and outer integuments and between nucellus and inner integument likely represent the first sign of ovule degeneration with exfoliated integuments [14]. The embryo sac of Sangiovese cv., as previously reported also for Gewürztraminer and Pinot noir [31], was fully developed at the onset of anthesis. Egg cell, central cell and synergids were observed in most of the samples, while antipodals (located at the chalazal end of the embryo sac) were not seen likely because they have a short life span and soon disintegrate [45]. Most of the Sangiovese ovaries inspected in this study presented the typical conformation described for the genus *Vitis*: bicarpelar, syncarpous (fused carpels into a unified compound gynoecium), and divided into two locules with two ovules each [27, 46]. However, we also observed some ovaries of Sangiovese presenting three locules with two ovules each, which evidences the presence of some flowers of Sangiovese with a tricarpelar, syncarpous ovary. This phenomenon had been previously observed in some ovaries of cultivated grapes [43, 45] and also, in mature berries of the wild species *Vitis labrusca* the reported number of hard well-developed seeds amounted to six [47]. Two types of syncarpous ovary ontogeny have been described based on the time of the carpel fusion event involved: congenital (carpels fused from the earliest emergence of their primordia) and post-congenital (fusion takes place during development). Syncarpy is congenital in 80% of angiosperms and it has also been reported as the main pathway for tricarpelar ontogeny in a Chinese *V. vinifera* cultivar, Xiangfei, with a high occurrence of tricarpelar flowers [43]. In our study, all locules of the three-locule ovaries seemed alike in size and morphology and contained two potentially functional ovules each, so it is likely that also in the case of the infrequent tricarpelar ovaries of Sangiovese a congenital type is involved; although further studies should be performed for confirmation.

## Conclusions

The histological sample preparation process we propose here can be used as a default procedure to obtain embedded ovaries or microscope slides that would require further procedures for examination (e.g. staining, in situ hybridization…). Safranin O—fast green FCF staining combination is an easy procedure and a valid staining for basic screenings about the presence of a normally and fully developed ovule and embryo sac in grapevine, which is therefore potentially functional. In addition, the proposed methodology could be potentially applied as a useful tool for studying the megagametophyte during flower development, as well as for comparative phenotyping of the embryo sac of seeded/seedless cultivars to get a clue about the underlying mechanisms of seedlessness, like defects during the female gametophyte development.

## Methods

### Reagents

- Ethanol (N. CAS 64-17-5).
- Formaldehyde (N. CAS 50-00-0).
- Glacial acetic acid (N. CAS 64-19-7).
- Xylene (N. CAS 1330-20-7).
- Chlorhydric acid (N. CAS 7647-01-0).
- Histoalcohol 99% (DIAPATH S.p.A.): ethanol-based alcohol mixture.
- Paraffin 56–58 °C (DIAPATH S.p.A.).
- Glycerinated albumen.

Covering medium for microscope slides and mounting medium for cover slips (Biomount BMT-100, Histo-line Laboratories).

### Solutions

- FAA fixative: proportions 10:50:5:35 of 37% formaldehyde: 95% ethanol: acetic acid: distilled water.
- Histoalcohol series: 95% histoalcohol diluted in distilled water to a concentration of 50, 70 and 95%.
- Ethanol series: 100% analytical grade ethanol diluted in distilled water to a concentration of 90, 70 and 50%.
- Safranin O (N. CAS 477-73-6) stock solution 1% (w/v) in 100% ethanol. It was diluted 1:1 in water to get the working staining solution.
- Fast Green FCF (N.CAS 2353-45-9) work solution 0.05% (w/v) in absolute ethanol.
- Acetic acid 1% (v/v) aqueous solution (it has to be fresh the day of the analysis).
- Acidulated 70% ethanol: addition of five drops of chlorhydric acid with a pasteur pipette of 7 mm diameter × 150 mm length in 250 mL of 70% analytical grade ethanol dilution.

### Equipment

- 250 mL plastic container with screw tap.
- 50 mL falcon tubes.
- Automated rotary microtome (Leica RM2245).
- Bell jar.
- Biopsy bags (DIAPATH S.p.A.).
- Biopsy cassettes (Histo-Line Laboratories).
- Compact oven ICT28 (FALC Instruments).
- Cryo console PF100 (Bio Optica).
- Disposable molds 15 × 15 and 24 × 24 mm (Histo-Line Laboratories).
- Dissection forceps.
- Embedding station DP500 (Bio Optica).
- Fume hood.
- Glass coverslips 24 × 24 mm.
- Glass staining dishes.
- Light microscope (Axio Lab A1, ZEISS).
- Microscope glass slides 26 × 76 mm.
- Microtome knives Patho Cutter II 35° 80 mm (DIAPATH S.p.A.).
- Round thermostatic bath BI (FALC Instruments).
- Slide drying bench MH6616 (Electrothermal).
- Stereomicroscope (Stemi 2000-CS, ZEISS).
- Vacuum pump.

### Plant material

Flower buds were collected from young plants of Sangiovese cultivar located in the grapevine germplasm collection of the Fondazione Edmund Mach (ITA362). Plant material was sampled from the same inflorescence at three phenological stages: E-L 15, E-L 17 and E-L 26 [32] in three different plants (Fig. 1).

### Sample collection and fixation (timing: 12 h fixation)

Groups of six to ten flowers were collected at each phenological stage keeping the pedicel. Immediately after sampling in the field, flowers were placed into a 50 mL falcon tube filled with FAA. In the laboratory, penetration of the fixative was facilitated by a vacuum infiltration for 30 min, or until the flowers sank to the bottom of the tube. An oil vacuum pump was connected to a bell jar vented into a fume hood and a pressure vacuum between − 0.7 and − 0.85 bar was applied. FAA volume should be at least 50× the volume of the plant material to be fixed. Then, plant material was fixed in FAA for at least 12 h at 4 °C. If material was not processed immediately, it could be conserved for a long period in fixative until dissection and subsequent sample processing.

### Dissection

Within 1 month of fixation, two flowers per inflorescence at each phenological stage were selected for dissection: one for longitudinal and another for transversal sectioning. Gynoecium of each flower was isolated by removing the calyptra and stamens (using a stereomicroscope and dissection forceps) and it was placed into a biopsy bag, which was then set into a biopsy cassette (previously labelled with the sample code). Cassettes in groups of maximum 12 were then immersed in a 250 mL container filled with FAA for storage and transported to the histology laboratory. The pedicel was conserved together with the gynoecium because it facilitated the orientation of the tissue sample when embedding it on paraffin.

### Sample dehydration and clearing (timing: 3 h 15 min dehydration + 45 min clearing)

The containing sample biopsy cassettes were transferred to a graded series of alcoholic solution. Sample dehydration was carried out at room temperature using an increasing graded histoalcohol series, from 50% to absolute histoalcohol, followed by a clearing step consisting of two changes of xylene (Table 2). Each dehydration and clearing step was performed in 500 mL of solvent, volume that could contain 12 biopsy cassettes at a time.

**Table 2:** Dehydration and clearing schedules within the histological preparation process of grapevine ovaries at pre-fruitset stages

Dehydration steps		
Histoalcohol grade	Change	Time
50%	1	15′
50%	2	30′
70%	1	15′
70%	2	30′
70%	3	30′
95%	1	15′
95%	2	30′
99%	1	30′

Clearing steps		
Solvent	Change	Time
Xylene	1	15′
Xylene	1	30′

### Sample infiltration and embedding (timing: (overnight + 5–6 h) infiltration + 30 min solidification)

Excess of xylene from the previous clearing step was removed by squeezing the cassettes on absorbent paper. Then, they were immersed in a glass staining dish filled with liquid paraffin and kept at 64 °C in a lab oven for tissue infiltration. One change of paraffin was done after overnight incubation. Incubation in the new paraffin continued for other 5–6 h. An embedding station was used for embedding samples in 15 × 15 or 24 × 24 mm disposable molds that were then set in a cryo console for fast paraffin solidification for at least 30 min. Two pistils per inflorescence per stage were separately embedded, one in longitudinal orientation and another in transversal orientation. Each sample was embedded in a paraffin block, getting as many paraffin blocks as samples processed.

### Sectioning with a rotary microtome

The paraffin block containing the sample was cooled on the cryo console before setting it on the rotary microtome. Sections or serial sections (ribbons) of 3–5 µm thickness were obtained. Each section or section ribbon was placed on the water surface of the paraffin floatation thermostatic bath (set at 37 °C) to which one drop of glycerinated albumen was previously added. In this way the section expands and flattens quickly. Subsequently, the section was affixed to a 26 × 76 mm microscope glass slide, which was then left in a slide drying bench (set about 5–10° below the melting point of the paraffin used) until completely dried.

### Staining of the slides and mounting (timing: 1 h 30 min deparaffination + 3 h 10 min staining + 25 min mounting per rack of 24 slides)

A safranin O—fast green FCF staining combination was used. Alternate slides for each sectioned pistil were stained. Before staining, slides were deparaffinized by incubating them for 30 min at 64 °C in a lab oven, then were placed immediately in xylene for 35 min (with one change of the solvent after 30 min) and consecutively slides underwent a series of ethanol of decreasing concentrations until 50% ethanol for tissue hydration (Table 3).

**Table 3:** Deparaffinization and hydration schedules for preparing slides to staining

Deparaffinization		
Solvent	Change	Time
Xylene	1	30′
Xylene	2	5′

Hydration		
Ethanol concentration	Change	Time
Absolute	1	5′
Absolute	2	5′
90%	1	5′
70%	1	5′
50%	1	5′

Immediately after deparaffinization sections were stained. The basic schedule proposed by Jensen [4] for safranin—fast green staining was set with some modifications:

- Stain 3 h in safranin O staining work solution.
- Wash out excess stain by immersing three times in distilled water.
- Dehydrate and differentiate safranin O for 1 min in acidulated 70% ethanol.
- Wash and dehydrate with 3 immersions in 90% ethanol.
- Finish dehydration with 3 immersions in 100% ethanol.
- Counterstain 1 min in fast green FCF staining solution.
- Fix and rinse excess fast green FCF by immersing 8 times in 1% acetic acid solution.
- Wash with 6 immersions in distilled water.
- Dehydrate with 6 immersions in 90% ethanol.
- Dehydrate with 8 immersions in 100% ethanol for two changes.
- Clear in xylene for two changes (10 s each).
- Mount the glass coverslip (24 × 24 mm) with mounting media one slide at a time.

### Observations at the light microscope and imaging processing

All the stained slides were observed through bright field microscopy. A digital camera (AxioCam ERc 5 s, ZEISS) was attached to the optical microscope and simultaneously connected to a computer. AxioVision Rel. 4.8 software (ZEISS) was used to examine and observe in “live” mode the samples, to get digital images and to do annotations on them. ImageJ software [48] was employed for image post-processing, which consisted of enhancing image contrasts and sharpness and performing customized images through the “custom montage plug-in”.

## Data Availability

Not applicable.
